# Variability in Executive Control Performance Is Predicted by Physical Activity

**DOI:** 10.3389/fnhum.2019.00463

**Published:** 2020-01-21

**Authors:** G. Kyle Gooderham, Simon Ho, Todd C. Handy

**Affiliations:** Attentional Neuroscience Laboratory, Department of Psychology, University of British Columbia, Vancouver, BC, Canada

**Keywords:** executive control, intraindividual variability, physical activity, exercise, young adults, cognitive load

## Abstract

Physical activity (PA) promotes neurogenesis and has neuroprotective effects on the brain, bolstering the structural and functional resources necessary for improved cognitive functioning. Intraindividual variability (IIV) in cognitive performance is linked to neuropsychological structure and functional ability. Despite evidence of the neurogenerative and neuroprotective effects of PA, limited investigation into the link between PA and IIV has been conducted. Across three studies we investigate the effect of PA on IIV in reaction time (RT) on three modified Flanker Tasks. The International PA Questionnaire was used to evaluate PA while the Attention Network Test (ANT) and two additional modified Flanker Tasks were used to assess executive control and attentional performance. RT coefficients of variation (RTCV) were calculated for each participant by dividing the standard deviation by the mean RT for each stimuli condition. Analysis revealed that basic RT was not associated with PA nor was PA predictive of IIV on the modified Flanker Tasks. However, three consistent findings emerged from analysis of the ANT. First, RTCV and moderate PA were positively related, such that more self-reported moderate PA was associated with greater IIV. Conversely, RTCV and vigorous PA were negatively related. Finally, when controlling for the effects of PA on IIV in young adults, variability decreases as age increases. In sum, PA is predictive of IIV on attentional and executive control tasks in young adults, though only at particular intensities and on certain tasks, indicating that task type and cognitive load are important determinants of the relationship between PA and cognitive performance. These findings are consistent with prior literature that suggests that the role of PA in young adults is reliant on specific interventions and measures in order to detect effects more readily found in adolescent and aging populations.

## Introduction

The enhancement of cognitive performance resulting from increased physical activity (PA) has been recognized for nearly five decades (Spirduso, 1975), and speculated for even longer (e.g., Burpee and Stroll, 1936; Pierson and Montoye, 1958). It is generally understood that physically active individuals, relative to their sedentary peers, demonstrate improved cognitive functioning on a multitude of cognitive tasks. However, research in young adult populations has been more equivocal. Combined evidence from studies investigating PA effects on cognitive functioning across the human lifespan suggests that these positive effects become stronger and more pronounced in the developing and aging brain, while those same effects are diminished or less robust in young adult populations (Voss et al., 2011; Hötting and Röder, 2013). Commonly utilized measures of executive control may not be sensitive enough to the neurological structural and functional alterations resulting from PA in young adult brains, which in turn may be contributing to the ambiguous findings. In this study we investigate whether within-subject reaction time (RT) variability in executive control, a relatively novel measure of cognitive performance within the PA-cognition literature, is responsive to PA levels in young adults and whether it may therefore provide evidence for the benefits of PA on cognitive function in this population.

Intense research effort has been placed on understanding the relationship between PA and cognitive function. Meta-analyses have demonstrated that a general positive association between increased PA and improved cognitive performance emerges in adult populations. For example, researchers have shown that PA habits that persist for longer than 12 months are related to improved executive control, memory, and processing speed in young to middle aged adults (Cox et al., 2016), while aerobic PA interventions of greater than 1 month have been associated with improved attentional and processing RT, executive control, and working memory in adult populations (Smith et al., 2010). Additionally, acute PA interventions, those occurring immediately preceding cognitive assessment, have been shown to benefit choice RT, executive control, and memory in young adults (Chang et al., 2012). Nevertheless, research has not universally endorsed PA as an effective intervention for enhancing cognitive performance, particularly for young adults (Etnier et al., 2006; Voss et al., 2011; Verburgh et al., 2014; Cox et al., 2016; Erickson et al., 2019).

Difficulties associated with eliciting and detecting effects of PA on cognition in young adults have led some to conclude that, while there are still positive effects, these effects are smaller and more sensitive to experimental characteristics (Guiney and Machado, 2013; Loprinzi and Kane, 2015). Others have gone further, suggesting that, because cognitive health is at its peak during young adulthood, it is unsurprising that PA has no discernable effect (Gourgouvelis et al., 2018). The foundational principle of these approaches is that if the brain is at maximum structural and functional integrity, then PA, an intervention that targets at least the neurological structure by way of neurochemical change in the brain that underlies the functional ability (Gomez-Pinilla and Hillman, 2013), should be ineffective.

A growing literature suggests that PA has profound effects on neuropsychological structure. Neuroimaging studies have shown that improved cognitive performance resulting from PA is linked to neurophysiological changes in hippocampal volume (Erickson et al., 2011), gray and white matter volume (Colcombe et al., 2006), and functional connectivity between brain regions (Voelcker-Rehage and Niemann, 2013). Additionally, researchers have identified the ways in which PA is a potent agent for inducing neurogenesis, neurotransmission, synaptogenesis, and angiogenesis (Vivar et al., 2012; Berchicci et al., 2013). PA has profound positive effects on neuroplasticity and thus brain health and cognitive performance (Colcombe et al., 2004; Hötting and Röder, 2013).

Variability is one indicator of neurological structural and functional integrity that is found embedded within nearly all measures of cognitive performance. However, most studies condense an individual’s performance into a single mean measure. Unfortunately, doing so discards a valuable indicator of performance, the variability. That is to say, summarizing performance as a mean value does not wholly capture performance on a task and discounts the uncertainty of that value as representative of the volatility in performance. The alternative is to capture the variability as its own measure that can then be compared across individuals or groups. Intraindividual variability (IIV) is a measure of a participant’s variability across trials and can be calculated from a single testing session or across multiple sessions. IIV is sensitive to structural and functional changes to neuroanatomy (MacDonald et al., 2006, 2009) and, recently, has been recognized as a measure of cognitive functioning that could therefore be responsive to PA. For example, researchers have shown that, while moderate and vigorous PA was not directly predictive of IIV on Stroop task RT, it was associated with increased rostral anterior cingulate cortex thickness which was related to RT IIV (Bento-Torres et al., 2019).

Our analysis seeks to replicate that conducted in the original study with IIV rather than basic RT as the variable of interest. We aim to determine whether IIV on an executive control task is responsive to self-reported PA levels in young adults and whether it provides a novel source of evidence for the benefits of PA on cognitive function. Two relationships inform our hypotheses. First, is the association between higher PA levels and better neurophysiological outcomes; and two, is the relationship between healthy neurophysiology and diminished IIV on cognitive tasks. Generally, we expect that as self-reported PA levels increase, IIV on executive control tasks will decline. Particularly, considering the volume of literature suggesting that the greatest benefits to cognitive performance emerge from moderate to vigorous intensity PA (Erickson et al., 2019), we expect that larger values of moderate to vigorous intensity PA will be associated with smaller IIV levels and, we do not expect low intensity PA to be related to IIV. Further, we anticipate that total METs and RTCV would be negatively related, such that as total METs increased, RTCV would lessen. Finally, given the positive relationship between individuals categorized as either active vs. sedentary according to the American College of Sports Medicine and cognitive control (Kamijo and Takeda, 2013), we expect that active rather than inactive participants will have smaller IIV. In order to test these hypotheses, we analyzed three data sets that were used to assess the relationship between PA and executive control in young adults (Ho et al., 2018).

## Materials and Methods

This article utilizes a dataset previously analyzed and reported (Ho et al., 2018). For further information regarding the purpose of the studies, their findings, and the implications consult Ho et al. (2018).

### Ethics and Participants

Ethical approval for the three experiments was provided by the University of British Columbia Behavioural Research Ethics Board. Participants were recruited from the University of British Columbia Department of Psychology’s Human Subject Pool and were remunerated with course credit. Individuals were considered eligible to participate in the studies if they were between the ages of 17 and 45 years, and were not physically disabled. Written informed consent was provided by each participant prior to the beginning of the experiments.

#### Experiment 1

A total of 267 participants were recruited as described above. Five participants were excluded from analysis due to computer malfunction or technical error and a further 14 were removed due to outlier rejection in accordance with the International PA Questionnaire (IPAQ) scoring protocol (The IPAQ Group, 2005). The final sample consisted of 248 participants (mean age = 20.49, SD = 2.70, 132 male).

#### Experiment 2

A total of 220 participants were recruited as described above. Two participants were excluded from analysis due to computer malfunction or technical error and a further 19 were removed due to outlier rejection in accordance with the IPAQ scoring protocol (The IPAQ Group, 2005). The final sample consisted of 199 participants (mean age = 20.04, SD = 1.76, 46 male).

#### Experiment 3

A total of 210 participants were recruited as described above. Four participants were excluded from analysis due to computer malfunction or technical error and a further 11 were removed due to outlier rejection in accordance with the IPAQ scoring protocol (The IPAQ Group, 2005). The final sample consisted of 195 participants (mean age = 20.32, SD = 2.72, 47 male).

### Procedure

Prior to the start of the experimental procedure participants provided written informed consent. Participants completed the IPAQ followed immediately by the cognitive testing.

### Apparatus

The cognitive tasks and questionnaires were displayed on 19^″^ LCD monitors at a display resolution of 1280 × 1024 using the Windows 7 operating system. The open-source Cognitive Battery 3.2 software package (Ho, 2015), which utilizes Python 3.6.4 and Pygame 1.9.3 for stimulus display, was employed for data collection.

### Measures

#### International Physical Activity Questionnaire

The long-form IPAQ is a self-administered PA survey that measures free-living PA over the antecedent 7 days (International Physical Activity Questionnaire, n.d.). The IPAQ has high validity and reliability (Craig et al., 2003; Hagströmer et al., 2006), and has been demonstrated to be stable and accurate in young adult populations (Dinger et al., 2006) as well as being robust to differences in age, sex, and language (Craig et al., 2003; Bauman et al., 2009; Wanner et al., 2016). Further, the IPAQ has been used as a measure of PA in numerous studies comparing the efficacy of PA on cognitive performance, including task switching (Kamijo and Takeda, 2010), spatial priming (Kamijo and Takeda, 2009), RT (Kamijo et al., 2009), response monitoring (Kamijo and Takeda, 2013), response inhibition (Wiedemann et al., 2014), and structural and functional neurophysiological connectivity (Kamijo et al., 2011).

The IPAQ measures across several domains of daily living, including PA related to an individual’s job, transportation, housework, house maintenance, caring for family, and recreation, sport, and leisure time. Participants report the daily totals of each activity and the number of days per week that they engage in that activity. Those values are then multiplied to produce a weekly total. Activities are categorized by type and intensity before being multiplied by metabolic equivalent of task (MET) values corresponding to the exertion required to complete the task (Ainsworth et al., 2000). METs are calculated as 1.0 (4.184 kJ) × kg^–1^ × h^–1^, and are used to assess the metabolic expenditure required to complete a task at a particular intensity, enabling for the comparison of energy consumption across PA types, durations, and intensities (Ainsworth et al., 2000). The scoring protocol provides for the calculation of total vigorous, moderate, and low intensity METs, along with total sitting time (The IPAQ Group, 2005). See Table 1 for PA METs for each experiment.

**TABLE 1:** Physical activity levels.

			Correlation coefficients		
	Mean	SD	1	2	3
**Experiment 1**					
1. Low intensity METs	1282.78	1365.87	–		
2. Moderate intensity METs	1072.53	1411.67	0.12	–	
3. Vigorous intensity METs	1373.87	1782.20	0.07	0.28^∗∗^	–
Total METs	3728.70	3045.97	–	–	–
**Experiment 2**					
1. Low intensity METs	2037.83	1776.44	–		
2. Moderate intensity METs	1104.16	1456.33	0.21^∗∗^	–	
3. Vigorous intensity METs	1359.24	2058.08	0.11	0.06	–
Total METs	4501.22	3437.42	–	–	–
**Experiment 3**					
1. Low intensity METs	2076.12	2000.06	–		
2. Moderate intensity METs	1243.81	1551.68	0.24^∗∗^	–	
3. Vigorous intensity METs	1623.80	2733.12	0.04	0.42^∗∗^	–
Total METs	4943.73	4399.06	–	–	–

*^∗∗^*p* < 0.01.*

#### Attention Network Test

The Attention Network Test (ANT) is a modified version of the Eriksen flanker task and assesses three aspects of attentional functioning (Fan et al., 2002). Executive control, alerting, and orienting of the attentional network are measured by calculating the difference in mean RT for four cue conditions and three flanker conditions. The stimuli presented are typical of the flanker task, where one central target arrow is flanked on either side by two distractor arrows, with the addition of a neutral condition where the flanking arrows are replaced by lines. The result is a row of five arrows. In the congruent condition the target arrow is either left or rightward facing and the flanking arrows share the same orientation. The incongruent condition differs in that the flanking arrows are oppositely oriented to the target arrow. The cues differ in the information that they provide to the participant in advance of the stimuli onset. In the no-cue condition a fixation cross is provided but no cue is given to warn of the imminent onset of the stimuli. The further three conditions alert the participant of the upcoming presentation of the stimuli. The central-cue replaces the fixation cross with a central asterisk, the double-cue places an asterisk both above and below the central fixation point, and the spatial-cue orients the participant to the location of the stimuli by providing an asterisk at the stimuli location. Unlike the typical implementation of the ANT, which involves calculating the difference between different cue and flanker conditions to determine executive control, alerting, and orienting scores, we calculated coefficients of variation values for each cue and flanker condition in order to directly measure IIV. A single trial began with the presentation of a fixation cross for 400–1600 ms, followed by the display of the cue for 100 ms. This was then followed by another fixation cross, displayed for 400 ms, after which the stimulus was presented for up to 1700 ms or until a response was recorded. Participants indicated the direction of the target arrow using the arrow keys on a keyboard. The RT was recorded in milliseconds as the time between the onset of the flanker stimulus and the response. Participants completed three sets of 96 trials, totaling 288 trials. Prior to testing, participants completed one practice block consisting of 24 trials.

#### Experiment 2 Eriksen Flanker Task

In Experiment 2, the Eriksen flanker task was used to determine executive control performance while limiting the length and complexity of the experiment to reduce fatigue effects for participants. Each trial opened with the presentation of a centered fixation cross for 1000 ms, which was subsequently replaced by the flanker arrowhead cue for 200 ms. The same four arrowhead configurations, leftward-congruent, leftward-incongruent, rightward-congruent, or rightward-incongruent, were used as stimuli in Experiment 2 as in Experiment 1. Participants were given 1500 ms to record a response. Next, feedback regarding the accuracy of performance on the trial was provided as either “correct,” “incorrect,” or “too slow.” Participants indicated the direction of the target arrow using the arrow keys on a keyboard. The RT was recorded in milliseconds as the time between the onset of the flanker stimulus and the response. The task began with 12 practice trials followed by 100 randomized and equiprobable stimuli presentations.

#### Experiment 3 Eriksen Flanker Task

Experiment 3 used an identical paradigm to Experiment 2 except for the addition of an incompatible condition. In the incompatible condition, participants were instructed to respond in the opposite direction to the central arrow via keyboard keypress. The introduction of the incompatible condition results in four measures: compatible-congruent, compatible-incongruent, incompatible-congruent, or incompatible- incongruent trials. The task began with 12 practice trials followed by 100 randomized trials with equiprobable congruency presentations. Compatible and incompatible trials were blocked and counterbalanced between participants.

### Coefficient of Variation

The RT coefficient of variation (RTCV) was selected as the primary measure of individual variability and was the dependent variable in data analysis. The RTCV is calculated by dividing the standard deviation of a participant’s RT by their mean RT for each measure. For example, in Experiment 3, the standard deviation of the RT for all of one participant’s incompatible-congruent trials would be divided by the mean RT of the corresponding trials. The resulting value is a standardized score that can be compared across measures. A RTCV was calculated for each participant for each cognitive measurement.

### Data Analysis

Three different coding methodologies were implemented to enable comparison with other literature as well as to demonstrate how the coding of PA may be determinate in evaluating the efficacy of the PA–cognition relationship. Model 1 utilized the IPAQ’s continuous scoring protocol, with total vigorous, moderate, and low intensity METs as the predictor variables (IPAQ Scoring Protocol - International Physical Activity Questionnaire, 2005). In Model 2, we calculated total weekly METs independent of intensity level. Finally, Model 3 used a scoring protocol that categorizes participants as either sedentary or active according to recommendations by the American College of Sports Medicine (Riebe et al., 2018). Participants are categorized as active if they report either ≥5 days/week of moderate intensity or ≥3 days/week of vigorous intensity PA, while inactive participants report ≤2 days/week of moderate or vigorous intensity PA.

Multiple regression was used to determine the relationship between PA, as measured by the IPAQ, and the coefficients of variation under each experimental condition. The covariates of participant age and sex were included in each regression model. Age, IPAQ METs, and RTCV scores were standardized prior to data analysis.

## Results

Original analysis of these data sets found no evidence to support that PA over the previous 7 days impacts basic RT on executive control tasks (Ho et al., 2018).

Correlational analysis was conducted to determine the relationships between low, moderate, and vigorous PA METs in each experiment. Significant positive correlations were observed between low and moderate PA METs in Experiments 2 and 3 as well as between moderate and vigorous PA METs in Experiments 1 and 3 (Table 1). Variance inflation factors were calculated for each model due to the potential for collinearity between predictors within the regression models. All variance inflation factors were within the acceptable range (<1.5).

Two between-subjects ANOVAs were conducted to determine the effect of experimental flanker task design on compatible-congruent and compatible-incongruent unstandardized RTs. There was a significant effect of flanker task design on compatible-congruent RT, *F*(2,639) = 208.3, *p* < 0.001, η^2^ = 0.40. A *post hoc* Tukey test indicated that compatible-congruent RT on the ANT was significantly greater than either other modified flanker task, *p*s < 0.001. However, the compatible-congruent RTs of Experiments 2 and 3 did not significantly differ, *p* = 1.00. Compatible-incongruent RT differed significantly by flanker task, *F*(2,639) = 332, *p* < 0.001, η^2^ = 0.51. Similarly, the compatible-incongruent ANT trials were significantly slower than those of Experiment 2 or 3, *p*s < 0.001, and RT was not significantly different between Experiments 2 and 3, *p* = 0.79.

Additional analysis was conducted on the differences between RTCV across Experiments 1–3. As with basic RTs, two between-subjects ANOVAs were conducted to analyze the effects of experimental flanker task on compatible-congruent and compatible-incongruent RTCV. There was a significant effect of flanker task on compatible-congruent RTCV, *F*(2,639) = 44.59, *p* < 0.001, η^2^ = 0.12. Compatible-congruent RTCV was significantly larger in the ANT than either other modified flanker task, *p*s < 0.001. In addition, RTCV was greater in Experiment 3 than Experiment 2, *p* = 0.004. On compatible-incongruent trials, we observed a significant effect of flanker task, *F*(2,639) = 49.33, *p* < 0.001, η^2^ = 0.13. Again, a *post hoc* Tukey test determined that compatible-incongruent RTCV was greater in the ANT than either other modified flanker task, *p*s < 0.001, while Experiment 3 RTCV was also larger than Experiment 2’s RTCV, *p* < 0.001. See Table 2 for the unstandardized RT as well as the RTCV values.

**TABLE 2:** Unstandardized reaction time in milliseconds and trial accuracy.

	Mean RT	SD RT	Mean RTCV	SD RTCV	Mean accuracy (%)	SD accuracy (%)
**Experiment 1**						
Neutral	559.12	81.06	0.205	0.094	98.59	4.32
Congruent	592.31	90.27	0.218	0.087	98.92	4.37
Incongruent	694.83	103.58	0.205	0.071	91.45	9.54
No cue	662.46	96.05	0.228	0.079	96.16	5.85
Center	612.26	89.37	0.227	0.083	95.75	5.72
Spatial	575.94	92.21	0.223	0.085	97.30	5.46
Double	611.02	87.42	0.224	0.075	96.07	5.52
**Experiment 2**						
Compatible-congruent	470.43	56.64	0.143	0.069	99.05	4.35
Compatible-incongruent	525.76	62.82	0.140	0.055	95.44	7.93
**Experiment 3**						
Compatible-congruent	470.20	65.23	0.171	0.095	98.89	2.81
Compatible-incongruent	520.29	67.88	0.169	0.079	94.49	7.07
Incompatible-congruent	505.01	70.38	0.173	0.075	97.62	3.48
Incompatible-incongruent	530.05	82.93	0.185	0.071	96.30	5.06

Trial accuracy was submitted to two between-subjects ANOVAs to determine if they differed by flanker task. Compatible-congruent accuracy did not vary by flanker task, *F*(2,639) = 0.09, *p* = 0.92, η^2^ = 0.00. However, there was a significant effect of flanker task on compatible-incongruent trial accuracy, *F*(2,639) = 14.13, *p* < 0.001, η^2^ = 0.04. Tukey’s *post hoc* comparison revealed that compatible-incongruent trial accuracy was significantly lower on the ANT than either other flanker task, *p*s < 0.001. Flanker task accuracy in Experiments 2 and 3 did not significantly differ, *p* = 0.50. Accuracy is reported in Table 2.

### Experiment 1

Seven measures of attentional and executive control function were created by isolating each flanker presentation of the ANT. Larger moderate intensity MET scores were predictive of increased RTCV on neutral, congruent, no cue, and double cue conditions. However, larger vigorous intensity MET values was predictive of decreased RTCV on neutral cue conditions. Participant age was associated with RTCV, such that as age increased, IIV decreased (Table 3).

**TABLE 3:** Physical activity intensity as predictors of RTCV on the Attention Network Test.

	Neutral cue		Congruent		Incongruent		No cue		Central cue		Spatial cue		Double cue	
Predictor	β	β	β	β	β	β	β	β	β	β	β	β	β	β
		95% CI		95% CI		95% CI		95% CI		95% CI		95% CI		95% CI
		[LL, UL]		[LL, UL]		[LL, UL]		[LL, UL]		[LL, UL]		[LL, UL]		[LL, UL]
Age	−0.14^∗∗^	[−0.27, −0.02]	−0.12^∗∗^	[−0.25, −0.00]	−0.11^∗^	[−0.24, 0.01]	−0.10	[−0.22, 0.03]	−0.12^∗^	[−0.25, 0.00]	−0.14^∗∗^	[−0.26, −0.01]	−0.13^∗∗^	[−0.26, −0.01]
Sex	0.03	[−0.22, 0.28]	−0.01	[−0.26, 0.24]	−0.02	[−0.28, 0.23]	−0.08	[−0.33, 0.18]	0.05	[−0.20, 0.31]	0.07	[−0.18, 0.32]	0.02	[−0.23, 0.28]
Low intensity METs	0.00	[−0.12, 0.13]	0.09	[−0.04, 0.22]	0.06	[−0.07, 0.18]	0.04	[−0.08, 0.17]	0.05	[−0.08, 0.17]	0.03	[−0.10, 0.16]	0.08	[−0.05, 0.20]
Moderate intensity METs	0.18^∗∗∗^	[0.05, 0.30]	0.17^∗∗^	[0.04, 0.29]	0.10	[−0.04, 0.23]	0.17^∗∗^	[0.04, 0.30]	0.09	[−0.04, 0.23]	0.12^∗^	[−0.01, 0.25]	0.15^∗∗^	[0.02, 0.28]
Vigorous intensity METs	−0.13^∗∗^	[−0.26, −0.01]	−0.09	[−0.22, 0.04]	0.07	[−0.20, 0.06]	−0.12^∗^	[−0.25, 0.01]	−0.07	[−0.20, 0.06]	−0.13^∗^	[−0.26, 0.00]	−0.05	[−0.18, 0.08]
*R*^2^	0.06^∗∗^		0.05^∗∗^		0.03		0.05^∗∗^		0.03		0.04^∗^		0.04^∗^	

*A significant β-weight indicates the semi-partial correlation is also significant. LL and UL indicate the lower and upper limits of a confidence interval, respectively. ^∗^*p* < 0.10; ^∗∗^*p* < 0.05; ^∗∗∗^*p* < 0.01.*

Attention Network Test RTCV was not predicted by total METs. Similarly, the categorization of participants according to the ACSM guide for weekly PA was not predictive of RTCV on any measures captured by the ANT. In both Models 2 and 3, participant’s age was predictive of neutral RTCV, spatial RTCV, and double RTCV while being marginally predictive of congruent RTCV, incongruent RTCV, and central RTCV (Table 4).

**TABLE 4:** Total physical activity METs and ACSM categorization as predictor of RTCV on the Attention Network Test.

		Neutral cue		Congruent		Incongruent		No cue		Central cue		Spatial cue		Double cue	
	Predictor	β	β	β	β	β	β	β	β	β	β	β	β	β	β
			95% CI		95% CI		95% CI		95% CI		95% CI		95% CI		95% CI
			[LL, UL]		[LL, UL]		[LL, UL]		[LL, UL]		[LL, UL]		[LL, UL]		[LL, UL]
**Model 2**															
	Age	–0.14^∗∗^	[−0.27, −0.02]	−0.12^∗^	[−0.24, 0.01]	−0.11^∗^	[−0.23, 0.02]	–0.09	[−0.22, 0.03]	−0.12^∗^	[−0.25, 0.01]	–0.13^∗∗^	[−0.26, −0.01]	–0.13^∗∗^	[−0.25, −0.00]
	Sex	0.03	[−0.22, 0.28]	–0.03	[−0.28, 0.22]	–0.04	[−0.29, 0.21]	–0.09	[−0.34, 0.16]	0.04	[−0.21, 0.29]	0.06	[−0.20, 0.31]	0.01	[−0.24, 0.26]
	Total MET	0.02	[−0.10, 0.15]	0.10	[−0.03, 0.22]	0.04	[−0.08, 0.17]	0.05	[−0.07, 0.18]	0.04	[−0.09, 0.16]	0.00	[−0.12, 0.13]	0.11	[−0.02, 0.23]
	*R*^2^	0.02		0.02		0.01		0.01		0.02		0.02		0.03.	
**Model 3**															
	Age	–0.14^∗∗^	[−0.27, −0.02]	−0.12^∗^	[−0.24, 0.01]	−0.11^∗^	[−0.23, 0.02]	–0.09	[−0.22, 0.03]	−0.12^∗^	[−0.25, 0.00]	–0.13^∗∗^	[−0.26, −0.01]	–0.13^∗∗^	[−0.25, −0.00]
	Sex	0.02	[−0.23, 0.27]	–0.03	[−0.28, 0.22]	–0.04	[−0.29, 0.22]	–0.09	[−0.34, 0.17]	0.04	[−0.22, 0.29]	0.05	[−0.20, 0.31]	0.01	[−0.24, 0.26]
	ACSM	0.10	[−0.17, 0.38]	0.08	[−0.19, 0.36]	–0.01	[−0.29, 0.26]	0.02	[−0.26, 0.29]	0.07	[−0.21, 0.34]	0.01	[−0.26, 0.29]	0.04	[−0.23, 0.31]
	*R*^2^	0.02		0.02		0.01		0.01		0.02		0.02		0.02	

*A significant *β*-weight indicates the semi-partial correlation is also significant. *LL* and *UL* indicate the lower and upper limits of a confidence interval, respectively. ^∗^*p* < 0.10. ^∗∗^*p* < 0.05.*

### Experiment 2

A paired samples *t*-test demonstrated no effect of congruent versus incongruent conditions on RTCV, *t*(198) = 0.70, *p* = 0.49.

Physical activity was not predictive of congruent or incongruent RTCV in the Eriksen flanker task according to any of the scoring protocols employed. However, sex was a significant predictor of congruent RTCV in all three models and was either a significant or a marginally significant predictor of incongruent RTCV in all three models (Table 5).

**TABLE 5:** Physical activity as predictors of RTCV on the Experiment 2 Eriksen flanker task.

	Model 1				Model 2				Model 3			
	Compatible-congruent		Compatible-incongruent		Compatible-congruent		Compatible-incongruent		Compatible-congruent		Compatible-incongruent	
Predictor	β	β	β	β	β	β	β	β	β	β	β	β
		95% CI		95% CI		95% CI		95% CI		95% CI		95% CI
		[LL, UL]		[LL, UL]		[LL, UL]		[LL, UL]		[LL, UL]		[LL, UL]
Age	–0.03	[−0.18, 0.11]	–0.11	[−0.25, 0.03]	–0.03	[−0.17, 0.11]	–0.11	[−0.25, 0.02]	–0.03	[−0.17, 0.11]	–0.11	[−0.25, 0.03]
Sex	0.4^∗∗^	[0.07, 0.74]	0.34^∗∗^	[0.01, 0.67]	0.41^∗∗^	[0.08, 0.74]	0.34^∗∗^	[0.01, 0.67]	0.41^∗∗^	[0.08, 0.75]	0.32^∗^	[−0.01, 0.65]
Low intensity METs	–0.03	[−0.18, 0.11]	0.08	[−0.07, 0.22]								
Moderate intensity METs	0.03	[−0.12, 0.17]	–0.04	[−0.18, 0.10]								
Vigorous intensity METs	–0.02	[−0.16, 0.12]	0.08	[−0.06, 0.22]								
Total METs					–0.02	[−0.16, 0.12]	0.09	[−0.05, 0.22]				
ACSM									0.03	[−0.25, 0.32]	–0.05	[−0.34, 0.24]
*R*^2^	0.03		0.04		0.03		0.04^∗^		0.03		0.03	

*A significant β-weight indicates the semi-partial correlation is also significant. LL and UL indicate the lower and upper limits of a confidence interval, respectively. ^∗^*p* < 0.10. ^∗∗^*p* < 0.05.*

### Experiment 3

A 2 (congruency: congruent RTCV, incongruent RTCV) × 2 (compatibility: compatible RTCV, incompatible RTCV) within-subjects ANOVA was used to confirm the manipulation of the congruency and compatibility conditions on the unstandardized RTCV scores. Following outlier rejection, described above, there was a significant interaction between compatibility and congruency, *F*(1,194) = 4.13, *p* = 0.043. Given the significant interaction, we conducted a Bonferroni corrected analysis of the simple main effects. On the compatible trials, there was no significant effect of congruency on RTCV (*p* = 0.67). However, for incompatible trials, there is a significant simple main effect of congruency (*p* = 0.18) such that RTCV was greater for incongruent trials in the incompatible condition. We observed no significant main effects of either the compatibility condition, *F*(1,194) = 2.69, *p* = 0.10, or the congruency condition, *F*(1,194) = 1.76, *p* = 0.19.

Neither PA METs by intensity nor total METs were predictive of RTCV of any of the four conditions. In Model 3, ACSM category was predictive of incompatible-congruent RTCV, β = −0.35, 95% CI [−0.65, −0.05], *t*(191) = 2.31, *p* = 0.022.

Sex predicted compatible-congruent CV in each model, Model 1: β = 0.35, 95% CI [0.01, 0.68], *t*(189) = 2.03, *p* = 0.044; Model 2: β = 0.34, 95% CI [0.01, 0.67], *t*(191) = 2.05, *p* = 0.042; Model 3: β = 0.35, 95% CI [0.02, 0.68], *t*(191) = 2.07, *p* = 0.039. Age did not predict RTCV in the compatible-congruent condition. Neither age nor sex was predictive of compatible-incongruent, incompatible-congruent, or incompatible-incongruent RTCV in Models 1–3.

## Discussion

The purpose of this secondary analysis was to investigate the link between PA levels in healthy, young adults, and IIV on attentional and executive control tasks. We hypothesized that, as PA levels increased, IIV would decline. Generally, our findings did not support our hypotheses. IIV on the flanker and the modified flanker task was not predicted by PA. Despite this, three consistent findings emerged from analysis of the ANT. First, RTCV and moderate PA were positively related, such that more self-reported moderate PA was associated with greater IIV. Conversely, RTCV and vigorous PA were negatively related. Finally, when controlling for the effects of PA on IIV in young adults, variability decreases as age increases. These results support three conclusions about the role of PA on IIV in cognitive functioning. First, the intensity of PA is predictive of IIV in attentional and executive control performance. Second, when controlling for the levels of PA in young adults, IIV decreased with increasing participant age. Lastly, task type and cognitive load are important determinants of the relationship between PA and cognitive performance, with IIV offering a novel measure of cognitive functioning.

Our findings confirm the predictive effect of PA intensity over the preceding 7 days on IIV in executive control tasks and attentional tasks. The intensity of PA interventions and their resultant outcome on cognitive function is a well-documented effect in acute PA literature. Moderate intensity activity seemingly optimally affects cognitive function (Brisswalter et al., 2002; Kashihara et al., 2009; Soga et al., 2015). Others report that intensity variably and selectively impacts cognitive function with low intensity activity providing the most immediate benefit but little long-term gain while vigorous intensity activity has the opposite effect (Chang et al., 2012). However, with adult populations, the PA intensity of aerobic training interventions that were greater than 1-month was not found to be predictive of attention or executive control (Smith et al., 2010). Our data suggest that PA intensity has differential effects on IIV in cognitive performance, at least during the 7 days preceding testing.

The positive association between moderate intensity PA levels and IIV is surprising and offers two diverging interpretations depending on how IIV is construed. The first is that moderate intensity PA negatively impacts cognitive performance by increasing variability. As mentioned previously, greater dispersion of RTs across task trials or types is attributed to diminished cognitive capacity while tighter clustering around the mean RT is indicative of cognitive stability. This interpretation is aligned with much of the literature on PA and IIV in which IIV is perceived as a negative consequence of poor physical fitness, health, or inactivity on cognitive performance. For example, investigations on the effect of obesity (Bauer and Houston, 2017; Chojnacki et al., 2018), aerobic fitness (Wu et al., 2011; Labelle et al., 2013; Moore et al., 2013; Bauermeister and Bunce, 2016), and PA (Gajewski and Falkenstein, 2015; Phillips et al., 2016; Fagot et al., 2019; Mehren et al., 2019; Wang et al., 2019) on IIV interpret decreased variability as the negative consequence of obesity, inactivity, and limited PA. However, cognitive functioning in the developing and aging process does not strictly adhere to this relationship. In developing brains, IIV suggests the pursuit of diverse exploratory strategies in solving complex tasks and is therefore an advantageous characteristic (MacDonald et al., 2009). Functional diversity and functional adaptability are two mechanisms by which an increase in IIV would demonstrate more robust cognitive performance, as an individual’s RT variability fluctuates according to strategy employment in the high cognitive demand tasks (Li et al., 2004). With this as our theoretical basis, we should not expect to see a relationship in low cognitive load conditions because the young adult brain is operating close to maximal function. Instead, only when under higher load, should we expect to see fluctuations in cognitive performance as a result of PA, with IIV signaling greater strategy exploration. The association between PA and IIV on the ANT but not on the flanker tasks may be supportive of this interpretation. While certainly no definitive claims can be made here with regards to either interpretation of IIV, our findings are, at minimum, cause for further investigation.

The type of cognitive task, and its cognitive load, are determinants of the relationship between PA and cognitive performance. The findings presented here further contribute to a significant body of evidence that suggests that PA differentially effects cognitive functions (e.g., Pérez et al., 2014; Swagerman et al., 2015; for a review, see Smith et al., 2010). However, our findings are distinct in that we demonstrate that variability in performance may be associated with the cognitive load of each trial, even within executive control tasks. Because IIV on congruent and incongruent flanker trials was not predicted by PA on either version of the modified Erikson flanker task, but congruent trial RTCV was predicted by PA intensity in the ANT, these findings are suggestive of the role of cognitive load on IIV. This interpretation is supported by the significantly larger mean basic RT, and mean RTCV of the ANT relative to either other modified flanker task. Furthermore, mean basic RT SD and RTCV SD were also generally higher for the ANT than for either version of the flanker task. These data suggest that the interaction between cue presentation and flanker congruency in the ANT elicits an effect not present in either version of the flanker task. Furthermore, the interspersion of task rules within blocks of trials on the ANT should increase cognitive load relative to the singular rule set in the blocked trials of the congruency or compatibility conditions on the modified flanker tasks. This interaction could be the increase in cognitive load due to orienting attention on trials where the flanker is not presented centrally.

Finally, we report that IIV decreased with increasing participant age when controlling for the levels of PA in young adults. This finding is surprising for three reasons. First, PA has, as described previously, more significant effects on the developing and aging brain (Hötting and Röder, 2013). Due the sample population in this study being rather unaffected by PA relative to younger and older populations investigated elsewhere, we would not anticipate age to emerge as a predictive factor. Second, the human brain is at its most developed in young adulthood (Craik and Bialystok, 2006) and therefore should be maximally structurally developed and functionally operating. Finally, is that IIV decreases through adolescence before stabilizing through young adulthood and then increasing again with advancing age (Hultsch et al., 2002; Williams et al., 2005, 2007; MacDonald et al., 2006). Because IIV plateaus in young adulthood, age is a surprising predictor in our models. Nonetheless, our data provide evidence for the role of increasing age in predicting less IIV in executive control when controlling for PA levels. These results could indicate that, even in young adulthood, age predicts IIV, and that PA levels mask the effect of age-related changes in IIV. However, given that the age of the participant population is relatively homogenous and centered around 20 years of age, we did not expect age to be predictive of IIV. In fact, we suspect the observed pattern may be a case of Simpson’s Paradox, wherein our data show this association but, in a general population this trend may disappear. Therefore, we are cautious in our interpretation and recognize the need to further investigate this finding in a larger, more age-diverse sample, before confirming this finding.

## Conclusion

In conclusion, self-reported PA is predictive of IIV on attentional and executive control tasks in young adults, though only at particular intensities and on subtypes of the assessments employed. These findings are consistent with prior literature which suggests that the role of PA in young adults is reliant on specific interventions and measures in order to detect effects more readily found in adolescent and aging populations. We provide evidence that variability in cognitive performance is responsive to PA in young adults and may prove to be a useful measure of cognitive functioning that to this point has been under-reported in the PA-cognition literature. Given the findings presented here, two research questions emerge. First, and of the utmost importance, is determining whether increased IIV as a result of PA in young adults serves an adaptive goal, and is therefore desired, or if it is indicative of declines in cognitive functional capacity. An evolutionary approach that emphasizes which cognitive domains would serve the greatest adaptive function as a result of increased aerobic fitness may prove fruitful. Finally, and related, is the necessity to establish if the findings presented here are generalizable across other cognitive domains. A greater understanding of which cognitive functions demonstrate changes to IIV as a result of PA may help inform how the cumulative short-term cognitive benefits of acute PA translate into long-term gains. Additional studies using randomized control trial paradigms will aid in better understanding the effects of PA on IIV.

## Data Availability Statement

The datasets analyzed for this study can be found at PLoS One: https://doi.org/10.1371/journal.pone.0209616.s001.

## Ethics Statement

The studies involving human participants were reviewed and approved by the University of British Columbia Behavioural Research Ethics Board. The patients/participants provided their written informed consent to participate in this study.

## Author Contributions

SH performed the study conceptualization and conducted the study. GG and TH came up with the idea for secondary analysis of variability within the original dataset. GG conducted the analysis of the data presented in this study and prepared the initial draft of the manuscript. SH and TH provided the editorial and intellectual contributions to the final manuscript.

## Conflict of Interest

The authors declare that the research was conducted in the absence of any commercial or financial relationships that could be construed as a potential conflict of interest.
