# Corrigendum: Variability in Executive Control Performance Is Predicted by Physical Activity

**DOI:** 10.3389/fnhum.2020.00115

**Published:** 2020-04-08

**Authors:** G. Kyle Gooderham, Simon Ho, Todd C. Handy

**Affiliations:** Attentional Neuroscience Laboratory, Department of Psychology, University of British Columbia, Vancouver, BC, Canada

**Keywords:** executive control, intraindividual variability, physical activity, exercise, young adults, cognitive load

In the original article, there was an error. The descriptive definition for the coefficient of variation was incorrectly provided. In the original manuscript the definition was given as the mean reaction time divided by the standard deviation. This was an error as the coefficient of variation was calculated as the standard deviation divided by the mean.

A correction has been made to the Materials and Methods section, subsection Coefficient of Variation, paragraph 1:

“The RT coefficient of variation (RTCV) was selected as the primary measure of individual variability and was the dependent variable in data analysis. The RTCV is calculated by dividing the standard deviation of a participant's RT by their mean RT for each measure. For example, in Experiment 3, the standard deviation of the RT for all of one participant's incompatible-congruent trials would be divided by the mean RT of the corresponding trials. The resulting value is a standardized score that can be compared across measures. A RTCV was calculated for each participant for each cognitive measurement.”

The authors apologize for this error and state that this does not change the scientific conclusions of the article in any way. The original article has been updated.

